# Could the use of agonist protocols benefit patients who do not respond well to human reproduction treatment?

**DOI:** 10.5935/1518-0557.20240057

**Published:** 2024

**Authors:** George Queiroz Vaz, Tereza Carolina Fonseca, Priscila Guyt Rebelo, Alessandra Evangelista, Maria Cecilia Erthal de Campos Martins, Paulo Gallo de Sá

**Affiliations:** 1 Department of Gynecology, UERJ - Rio de Janeiro State University, 20551-030 Rio de Janeiro, RJ, Brazil; 2 Vida Fertility Center - FertGroup, 22793-080 Rio de Janeiro, RJ, Brazil; 3 UERJ - Rio de Janeiro State University’s master student, 20551-030 Rio de Janeiro, RJ, Brazil

**Keywords:** infertility, female infertility, assisted reproduction, human reproduction, poor responder

## Abstract

**Objective:**

The primary goal of this article is to analyze whether there is still room for ovarian stimulation in poor responders prescribed the long protocol.

**Methods:**

This retrospective cohort study analyzed the medical charts of patients seen at the Vida Centro de Fertilidade, a private fertility clinic in Rio de Janeiro, Brazil, from January 2018 to June 2023. It included poor responders described based on the Bologna criteria who were first prescribed conventional treatment with an antagonist protocol, without success, and then the long agonist protocol. Statistical analysis was performed on the Statistical Package for the Social Sciences (version 20). Comparisons of continuous variables between groups were performed with the Mann-Whitney U test or Student’s t-test, as appropriate. The chi-square test was used to compare categorical variables. Statistical significance was achieved when p<0.05.

**Results:**

We found a better response among patients on the agonist than on the antagonist protocol in terms of number of follicles larger than 14 mm on the day of trigger (3.17 versus 2.1; p<0.05), number of eggs on the day of retrieval (3.5 versus 1.37; p<0.05), number of mature eggs (2.67 versus 1.37; p<0.05), and number of embryos after fertilization on the first day of development (1.87 versus 0.8; p<0.05). This protocol’s cancellation rate was slightly lower (0.03 versus 0.43; p<0.05).

**Conclusions:**

The long protocol still yields positive results in poor responders who were previously prescribed the antagonist protocol.

## INTRODUCTION

Poor responders are patients who, for some reason, have a low oocyte count for their age, fewer oocytes than indicated in previous ovary analysis, or a low ovarian reserve after assisted reproduction treatment. Treating these patients remains a challenge in assisted reproduction, as most of them not only have a small oocyte count after follicular puncture but also produce low-quality embryos after fertilization and an increased rate of canceled stimulation cycles (Patrizio *et al.*, 2015).

The main terms used to describe them include premature ovarian insufficiency, impending premature ovarian failure, or poor ovarian response. In over half of these patients, the etiology remains unidentified. Advanced age is the primary documented factor. It is accepted that a woman is born with a finite number of oocytes for her entire reproductive life, which declines with each menstrual cycle. However, several other etiologies exist, including genetic, metabolic, enzymatic, iatrogenic, toxic, autoimmune factors, and infectious diseases. (Blumenfeld *et al*., 1993; Blumenfeld, 2009, 2011; Cedars, 2022). Although the most successful treatment is donor egg implantation, most infertile women still prefer to try other therapies despite their low chances of success.

The prevalence of poor treatment response ranges from 10% to 24% (Patrizio *et al.*, 2015). Two validated classifications in the literature are used to define poor response. Previously, authors differed in the choice of treatment protocols for poor responders because there was no universal classification. The first consensus statement on the subject was published in 2010 by the ESHRE (Patrizio *et al.*, 2015), in which the Bologna criteria was introduced. According to these criteria, women described as poor responders must meet at least two of the following criteria: (1) history of poor ovarian response (POR), defined as having produced three or fewer oocytes with conventional stimulation protocols. (2) Having an antral follicle count (AFC) under 5 to 7 follicles or an anti-Müllerian hormone (AMH) level below 0.5-1.1 ng/ml. (3) advanced age, defined as being 40+ years old and having other risk factors for suboptimal ovarian response, such as prior ovarian surgery, genetic anomalies, radiotherapy, chemotherapy, or autoimmune diseases (Cedars, 2022).

Published in 2016, the POSEIDON (Patient-Oriented Strategies Encompassing Individualized Oocyte Number) optimized the Bologna criteria and proposed treatment and prognostic strategies for patients in various circumstances. (Humaidan *et al*., 2016).

Ovarian stimulation is an assisted reproduction procedure prescribed to patients with infertility or who wish to preserve future fertility. In this procedure, patients take gonadotropins to stimulate follicular growth and maximize the pool of available oocytes. The greater the number of follicles above 18mm, the greater the chances of obtaining mature oocytes and, therefore, the greater the chances of receiving an embryo after fertilization.

Achieving the highest number of follicular pools is a complex task. Numerous factors influence their recruitment, such as patient characteristics and the choice of medications by the human reproduction professional. Some patients, especially those of older age, respond poorly (Patrizio *et al.*, 2015).

Strategies have been described to attain the best follicular pool in patients with a low ovarian reserve. In 2003, Baerwald *et al.* (2003a; 2003b) described the existence of two or three follicular waves in the period between ovulations in healthy women. With that in mind, a new stimulation option was developed for these patients. Double stimulation aims, in the same menstrual cycle, to stimulate the growth of the oocyte pool both in the follicular and luteal phases (DuoStim). Double stimulation is designed to optimize the use of recruited follicles, including the smallest ones, which suffered from slower or asynchronous growth during the first stimulation cycle. (Haahr *et al*., 2019; Polyzos and Drakopoulos, 2019; Sunkara *et al*., 2020).

According to Patrizio *et al.* (2015), the most used protocols for poor responders following the Bologna classification are the antagonist protocol (53%), the short protocol with a GnRH agonist (gonadotropin-releasing hormone) (20%), the microdose flair protocol with a GnRH (15%), and the long protocol with a GnRH agonist (9%).

New strategies for poor responders have emerged since the publication of the POSEIDON classification (Humaidan *et al*., 2016), including the use of a higher dose of gonadotropins (above 225IU up to 300IU), the combination of different types of gonadotropins (LH and FSH), the short and ultra-short protocols, the administration of microdoses of GnRH agonists (in an attempt to increase hormone flair up), a combination of the ultra-short GnRH agonist protocol with multiple doses of the antagonist protocol, the delayed-start gonadotropin-releasing hormone antagonist protocol, the mild stimulation protocol, which includes low doses of gonadotropins combined with oral stimulants or modified natural cycles, the pre-blocking protocol with a GnRH antagonist, and dual or double triggering, to name a few.

Other less-studied approaches have yet to gain scientific recognition. One is the use of pre-stimulation medications (also called priming) such as testosterone, dehydroepiandrosterone (DHEA), and growth hormone (GH). Such approaches aim to increase the receptors of FSH and GH in the granulosa cells, improving the recruitment of pre-antral follicles and the follicular growth of antral follicles. Some medications, such as contraceptives, may decrease the effect of priming.

Despite the strategies described in the literature, the stimulation approach has yet to emerge as the most suitable for poor responders. Customized controlled ovarian hyperstimulation appears to be an exciting option, but there is no consensus over the best means to achieve it. The treatment with GnRH agonists was replaced by the GnRH antagonist protocol and its variations (mild stimulation, etc.) since it causes a more significant blockade of the hypothalamic-pituitary-ovarian axis with fewer medications and for a lower cost, without increasing serum estradiol levels and thus reducing the risk of ovarian hyperstimulation syndrome (Duan *et al*., 2023). However, the antagonist protocol still delivers a final follicular count below expected in poor responders.

Until 2020, both protocols were considered equivalent for poor responders. However, recent studies have indicated otherwise. In a meta-analysis, patients described via the Bologna criteria were observed to have lower cancellation rates and higher clinical pregnancy rates when the agonist protocol was used compared to the antagonist protocol.

The use of carefully titrated GnRH agonists in patients with a low ovarian reserve and a history of hormonal response in previous treatments under controlled stimulation with gonadotropins may be beneficial. This study hypothesizes that, in the long protocol, the longer time of axis blockade may homogenize the follicular cohort and that more extended time on gonadotropins may expand the final follicular cohort. It analyzes whether the protocol with GnRH agonists still has room for patients with a poor ovarian response.

In this study, the agonist protocol is compared with the antagonist protocol in poor responders to correlate indices such as the number of eggs in follicular puncture, mature eggs, and fertilization rate, among others. This study aims to analyze whether the agonist protocol is still helpful in the array of strategies described in the literature for poor responders.

## MATERIALS AND METHODS

This retrospective cohort study analyzed the medical charts of patients seen at the Vida Centro de Fertilidade, a private fertility clinic in Rio de Janeiro, Brazil, from January 2018 to June 2023. It included poor responders described based on the Bologna criteria who were first prescribed conventional treatment with an antagonist protocol, without success, and then the long agonist protocol.

Ovarian stimulation and laboratory results were compared between the two protocols using participant data collected from electronic medical charts. The collected and analyzed variables included the following nominal numerical variables: number of days on the antagonist protocol, number of days on the agonist protocol, total treatment dose, number of larger follicles in both protocols, number of oocytes retrieved, number of mature oocytes, number of fertilized oocytes, number of transferred embryos, numbers of cycle cancellations (due to the absence of follicular growth or monofollicular growth), all in both protocols. After initial analysis, the data were summarized into length of stimulation in days, total dose of gonadotropins used, number of follicles larger than 14 mm on trigger day, number of oocytes retrieved, number of mature oocytes, number of embryos, number of cycle cancellations, and fertilization rate (%) in the antagonist and agonist protocols.

The conventional antagonist protocol involves ovarian stimulation with gonadotropins initiated in variable doses on days 2-3 of the menstrual cycle, determined based on patient age, ovarian reserve, and/or ovarian response to previous cycles. The dose was adjusted according to serum estradiol levels and serial vaginal ultrasound follicular diameter measurements. The application of the GnRH antagonist (0.25 mg/day, Cetrotide, or Orgalutran) was initiated when a follicle reached 13 mm and/or serum estradiol levels exceeded 400 pg/mL. A trigger was used to induce follicular maturation when at least two follicles reached an average diameter of 18mm. The ovarian puncture was performed 36 hours after the administration of recombinant human chorionic gonadotropin (Ovidrel).

In the long protocol with a GnRH agonist, Gonapeptil was administered daily, starting in the previous mid-luteal phase. After blocking the hypothalamus-pituitary-ovarian axis, which was confirmed by finding follicles measuring less than 10 mm on ultrasound examination, an endometrium measuring less than 5mm, or serum estradiol levels below 50pg/ml, gonadotropins were started in variable doses, depending on the patient’s age and/or ovarian response to previous cycles. The dose was adjusted according to serum estradiol levels and vaginal ultrasound follicular diameter measurements. Triggering induced follicular maturation when at least two follicles reached an average diameter of 18mm. The ovarian puncture was performed 36 hours after the administration of recombinant human chorionic gonadotropin (Ovidrel).

Statistical analysis was performed on the Statistical Package for the Social Sciences software (version 20). The Mann-Whitney U or Student’s t-test was used to compare continuous variables between the two groups, as appropriate. The chi-square or Fisher’s exact test was used to compare categorical variables. Statistical significance was defined as *p*<0.05 for all comparisons.

The local ethics committee approved the study. Informed consent was waived as it involved secondary data analysis of medical records.

## RESULTS

A total of 30 patients, ranging in age from 31 to 44 and with an average age of 38.1 years, were included. The mean anti-Müllerian hormone level was 0.55, and the mean body mass index (BMI) was 28.33. Table 1 describes the leading causes of infertility in the group, along with other relevant information. All patients were categorized as poor responders according to the Bologna criteria. They had undergone at least one in vitro fertilization cycle with the antagonist protocol.

**Table 1:** Patient Characteristics and Primary Infertility Factor.

Characteristics	Mean
Age	38.1
BMI^*^	28.33
AMH^**^	0.55
**Infertility Factor**	**N (%)**
Ovarian Insufficiency	8 (60%)
Endometriosis	9 (30%)
Male Factor	3 (10%)

* BMI (Body Mass Index)

** AMH (Anti-Müllerian Hormone).

As shown in Table 2, the statistical analysis using the chi-square test revealed that most of the results from the agonist and antagonist protocol treatments were statistically different. However, the total dose of gonadotropins used in these treatments was similar. Although not statistically significant, the difference indicated a trend toward higher doses in the agonist protocol. Differences with a *p*-value of less than 0.05 were considered statistically significant.

**Table 2:** A comparison of the agonist and antagonist protocols in poor responders.

Variable	Treatment		p-value
	Antagonist µ±SD	Agonist µ±SD	
Days of ovarian stimulation	11.13±2.99	13.73.56	0.003
Dose (UI)	3245.83±907.81	3355.3±1268.38	0.69
Total number of follicles >14mm on trigger day	2.1±1.82	3.17±2.22	0.02
Number of oocytes retrieved	1.87±1.99	3.5±3.22	0.01
Number of mature oocytes retrieved	1.37±1.56	2.67±2.72	0.02
Number of embryos on the first day (2PN)	0.8±1.15	1.87±2.34	0.02
Cycle cancellation	0.43±0.50	0.03±0.18	<0.001

µ±SD: Mean ± Standard Deviation.

Patients treated with the agonist protocol exhibited a better response, as evidenced by the higher numbers of follicles measuring more than 14 mm on trigger day, oocytes retrieved on puncture day, mature oocytes (MII), and increased embryo yield after fertilization on the first day of in vitro development (2PN). All these variables were statistically different. The detailed results can be found in Table 2.

Another part of the analysis focused on cancellation rates. Across all protocols, cancellation rates were meager. However, with the agonist protocol, the ovarian stimulation cancellation rate was close to zero, eliciting another statistically significant difference between the protocols. Table 2 shows the observed results.

Fertilization rates were compared between treatments using the agonist and antagonist protocols. The fertilization rate is a mathematical formula in which the number of embryos is divided by the number of mature follicles obtained after follicular aspiration. Although without statistical significance, the long protocol yielded higher fertilization rates than the antagonist protocol (58% *vs*. 69%, *p*=0.2). Further details can be found in Table 3.

**Table 3:** Fertilization Rates After Treatment with the Antagonist and Agonist Protocols.

Treatment	Antagonist	Agonist	p-value
Fertilization Rate (%)	58	69	0.24

## DISCUSSION

Despite significant advancements in assisted reproduction, ovarian stimulation in patients with diminished ovarian reserve remains a challenge. Poor ovarian response is characterized by ovarian insensitivity to both endogenous and exogenous hormones, resulting in reduced follicular recruitment, fewer mature oocytes retrieved, and a lower rate of high-quality embryos. Interestingly, despite the lower rate of high-quality embryos, there is no difference in pregnancy rates following embryo transfer between young, poor responders and patients with a normal ovarian response (De Sutter & Dhont, 2003).

According to the literature, some causes may be associated, such as FSH insensitivity, a shortened follicular phase, and asynchrony of follicle development (Oudendijk *et al.*, 2012). The main accepted explanation is poor response to FSH, where follicles express their receptors at different points in the menstrual cycle, resulting in follicular growth asynchrony. However, further elucidation and more studies are required (McGee & Hsueh, 2000). Another theory is that the irregular response to FSH is physiological and causes the recruitment of the dominant follicle in each cycle. Other acquired causes for low reserve include previous ovarian surgery, endometriosis, and post-infection adhesions - these would be explained by the reduction in ovarian flow, which thus reduces ovarian recruitment, in addition to smoking, inflammatory diets, and autoimmune disease (De Sutter & Dhont, 2003).

The difference between patients with low ovarian reserve is the FSH surge that occurs in the luteal phase of the previous cycle, which might explain a discrepant follicular cohort and an irregular oocyte response. Therefore, a protocol initiated in the follicular phase aims to block the early FSH surge in these patients, recruiting the maximum number of available follicles (Yang *et al.*, 2021).

Therefore, the agonist protocol would solve early recruitment and asynchronous follicular cohorts since pituitary blockade would start before the initial follicular phase. As a result, exogenous stimulation with gonadotropins becomes equitable and individualized. Furthermore, it is possible to choose the type of gonadotropin and the necessary dose, which can be adjusted during treatment (Orvieto *et al*., 2021). The major drawback of this protocol lies in the increase in the days of stimulation and, consequently, the total dose of gonadotropins (Ubaldi *et al*., 2016).

Our study found a slight increase in gonadotropin doses and length of stimulation in the agonist protocol, which can be explained by the more intense hypothalamic blockade required.

Therefore, the agonist protocol tends to be less affordable than the antagonist protocol. The difference stems from an additional 2.57 days of treatment and 109.43 IU of gonadotropins with the agonist protocol. The gonadotropin dose was comparatively more diluted and slightly higher in the agonist than in the antagonist protocol. The difference in cost exists but does not substantially affect the final price of treatment.

Cancellation of ovarian stimulation cycles occurs somewhat often in poor responders since they suffer from a lack of physiological response derived from ovaries without enough follicles to supply. According to a meta-analysis published in 2021 in which the outcomes of the two protocols were analyzed, the agonist protocol yielded lower cancellation rates than the antagonist protocol (Baka *et al.*, 2006). These findings resonate with our study, which found fewer cycle cancellations in patients treated with the agonist protocol. Although not statistically significant, probably due to the limited size of the population analyzed, a lower cancellation rate can be inferred in the agonist protocol. More studies with an adequate population size are needed to confirm these findings.

Analyzing the results of the agonist treatment, a higher number of follicles above 14mm was observed at the end of treatment, culminating in a higher number of retrieved oocytes and, therefore, a higher number of mature oocytes and fertilized embryos at the end of treatment. These findings align with most meta-analyses published from 2010 to 2021, in which no statistical differences were described between the two protocols (Pu *et al.*, 2011; Papamentzelopoulou *et al.*, 2021), indicating that more robust studies are needed. Achieving more viable embryos ultimately increases the number of embryo transfers. The more viable embryos are available to poor responders, the greater the chances of embryo implantation, pregnancy, and live births.

Higher fertilization rates were verified in favor of the agonist protocol in patients with a low ovarian reserve. This factor potentially improves the number of embryo transfers and the chances of pregnancy.

Due to its retrospective observational nature, the present study faces a few biases. Furthermore, we could not compare pregnancy rates between the protocols since the first treatment with an antagonist protocol did not yield embryos. Therefore, we could not analyze pregnancy rates using this protocol. Another area for improvement is the reduced size of the study population, which included only Brazilian patients, which may decrease the study’s applicability to other populations.

The study indicated that the agonist protocol is a good choice for poor responders since it increases the number of retrieved follicles, mature follicles, fertilized embryos, and the fertilization rate.

Poor responders undergoing assisted reproduction are aware of their lower chances of achieving pregnancy. The long protocol provides an additional opportunity for couples to become pregnant and have a baby.

## CONCLUSION

The success of the antagonist protocol has limited the use of the agonist protocol. However, the latter slightly decreases the overall cost of treatment and produces similar results in live births.

Poor responders benefit from the agonist protocol in areas such as the number of oocytes retrieved, mature eggs, and the fertilization rate.

The numerous limitations of this study, which include the small population size, its retrospective observational nature, enrolling only Brazilian patients, and using the Bologna classification, may make it difficult to reduce biases. To confront them, new studies, including larger populations, are needed to confirm the viability and superiority of the agonist protocol.
